# Immunosuppressives and biologics during pregnancy and lactation: A consensus report issued by the Austrian Societies of Gastroenterology and Hepatology and Rheumatology and Rehabilitation

**DOI:** 10.1007/s00508-019-1448-y

**Published:** 2019-01-14

**Authors:** Antonia Puchner, Hans Peter Gröchenig, Judith Sautner, Yvonne Helmy-Bader, Herbert Juch, Sieglinde Reinisch, Christoph Högenauer, Robert Koch, Josef Hermann, Andrea Studnicka-Benke, Wolfgang Weger, Rudolf Puchner, Clemens Dejaco

**Affiliations:** 1 Division of Rheumatology, Third Medical Department, Medical University of Vienna/Vienna General Hospital, Vienna, Austria; 2 Medical Department, Hospital of the Brothers of Mercy, St. Veit an der Glan, Austria; 3 Second Medical Department, Korneuburg-Stockerau Hospital/Lower Austrian Center for Rheumatology, Stockerau, Austria; 4 Department of Obstetrics and Gynecology, Medical University of Vienna, Vienna, Austria; 5 Department of Cell Biology, Histology and Embryology, Medical University of Graz, Graz, Austria; 6 Division of Gastroenterology and Hepatology, Department of Medicine III, Medical University of Vienna, Vienna, Austria; 7 Division of Gastroenterology and Hepatology, Medical Department, Medical University of Graz, Graz, Austria; 8 Division of Gastroenterology, First Medical Department, Medical University of Innsbruck, Innsbruck, Austria; 9 Division of Rheumatology and Immunology, Medical Department, Medical University of Graz, Graz, Austria; 10 Third Medical Department, Paracelsus Medical University, Salzburg, Austria; 11 Department of Dermatology and Venereology, Medical University of Graz, Graz, Austria; 12 Division of Rheumatology, Department of Medicine III, Medical University of Vienna, Vienna, Austria

**Keywords:** Autoimmune disease, Childbearing, Breastfeeding, Prescribing, TNF-α inhibitors, DMARDs

## Abstract

An increasing and early-onset use of immunosuppressives and biologics has become more frequently seen among patients with inflammatory bowel diseases (IBD) and rheumatic disorders. Many women in their childbearing years currently receive such medications, and some of them in an interdisciplinary setting. Many questions arise in women already pregnant or wishing to conceive with respect to continuing or discontinuing treatment, the risks borne by the newborns and their mothers and long-term safety. Together with the Austrian Society of Rheumatology and Rehabilitation, the IBD working group of the Austrian Society of Gastroenterology and Hepatology has elaborated consensus statements on the use of immunosuppressives and biologics in pregnancy and lactation. This is the first Austrian interdisciplinary consensus on this topic. It is intended to serve as a basis and support for providing advice to our patients and their treating physicians.

## Introduction

**Fig. 1:**
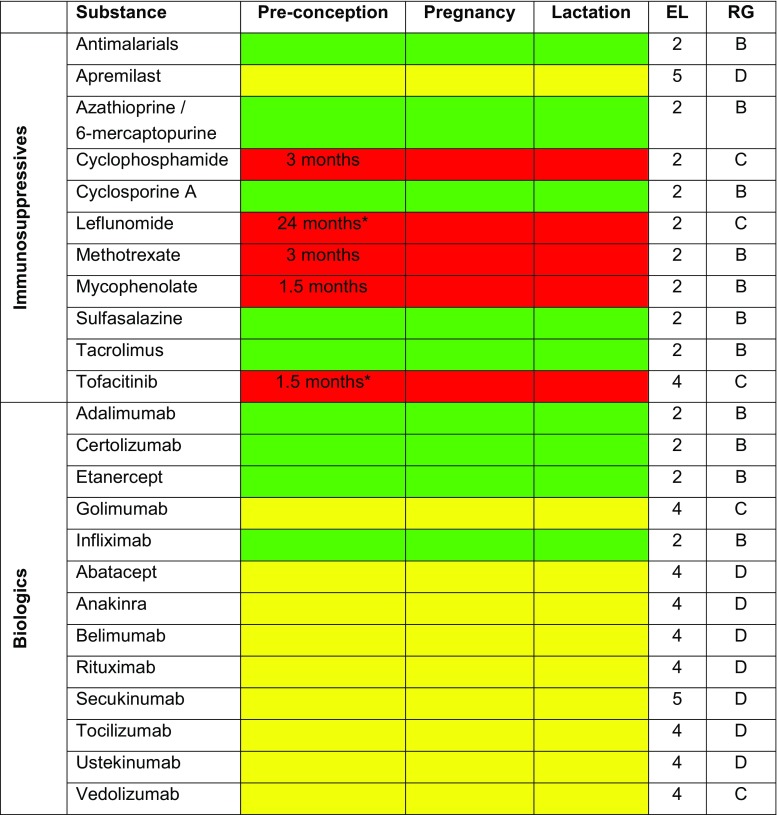
Substances and consensus recommendations regarding substance application preconception, during pregnancy and during lactation, including timing of preconception treatment discontinuation in months, levels of evidence and grades of recommendation (reference to pregnancy). (Recommendations: *green*, substance may be applied; *yellow*, data is insufficient for substance recommendation; *red*, substance application is not recommended. *EL* level of evidence, *RG* grade of recommendation. *Shown to be teratogenic in animal models, insufficient or unavailable data in humans)

The present consensus report issued by the Austrian Society of Gastroenterology and Hepatology (Österreichische Gesellschaft für Gastroenterologie und Hepatologie; ÖGGH) and the Austrian Society of Rheumatology and Rehabilitation (Österreichische Gesellschaft für Rheumatologie und Rehabilitation; ÖGR) is intended to provide practical guidelines for the application of immunosuppressives and biologics in pregnancy and the lactation period. The contributors to this consensus, who are also the authors of this report, drafted its text body and recommendations regarding the medications currently in use (Delphi process). All consensus participants are members of their respective specialist societies and have many years of experience in the treatment of outpatient and inpatient subjects with immunosuppressives and biologics. This body of expertise was complemented by three specialists in gynecology, dermatology and embryology, respectively. The recommendations and text are based on the available literature (systematic literature review by means of MEDLINE/PubMed, the Cochrane Database and abstracts from relevant international conferences) and were scrutinized according to the levels of evidence (EL) and grades of recommendation (RG) set forth by the Oxford Centre for Evidence-Based Medicine (Table 1). The consensus participants elaborated the statements and put them to vote on the occasion of a meeting on 13 October 2016. A statement was considered consented if it received 80% or more approval. Prior to printing, the statements and references were updated according to the current status of the literature (July 2018). The individual participants composed and edited the chapters of the consensus report, and all participants approved the final version. The substances and consensus recommendations regarding substance application preconception, during pregnancy and during lactation were summerized in Fig. 1.

**Table 1:** Levels of evidence and grades of recommendation set forth by the Oxford Centre for Evidence-Based Medicine [117]

*Levels of evidence (treatment benefits)*	
1^*^	Systematic review of randomized trials or *n*-of-1 trials
2^*^	Randomized trial or observational study with dramatic effect
3^*^	Non-randomized controlled cohort/follow-up study^**^
4^*^	Case-series, case-control studies, or historically controlled studies^**^
5^*^	Mechanism-based reasoning
*Levels of evidence (common treatment harms)*	
1^*^	Systematic review of randomized trials, systematic review of nested case-control studies, *n*-of-1 trial with the patient you are raising the question about, or observational study with dramatic effect
2^*^	Individual randomized trial or (exceptionally) observational study with dramatic effect
3^*^	Non-randomized controlled cohort/follow-up study (post-marketing surveillance) provided there are sufficient numbers to rule out a common harm. (For long-term harms, the duration of follow-up must be sufficient)^**^
4^*^	Case-series, case-control, or historically controlled studies^**^
5^*^	Mechanism-based reasoning
*Grades of recommendation*	
A	Consistent level 1 studies
B	Consistent level 2 or 3 studies *or* extrapolations from level 1 studies
C	Level 4 studies *or* extrapolations from level 2 or 3 studies
D	Level 5 evidence *or* troublingly inconsistent or inconclusive studies of any level

^*^Level may be graded down on the basis of study quality, imprecision, indirectness (study PICO does not match questions PICO^***^), because of inconsistency between studies, or because the absolute effect size is very small; levels may be graded up if there is a large or very large effect size

^**^As always, a systematic review is generally better than an individual study

^***^PICO (**P**atient, **I**ntervention, **C**omparison, **O**utcome)

Anti-inflammatory immunosuppressive (long-term) therapy remains a particular challenge to women in their childbearing years. A considerable number of treatment options and medications have become available, which may substantially ameliorate patients’ quality of life. Consequently, family planning among women under immunosuppressive therapies has increasingly gained in importance over the past years [1]. Substances such as 5‑aminosalicylic acid (5-ASA) and antimalarials have long become established treatments in pregnancy and lactation; however, the degree of information concerning the administration of novel immunosuppressive medications in gestation is often insufficiently complete to carry out precise embryotoxicological risk assessment [2]; however, it should be noted that most immunosuppressive therapies in pregnancy are acceptable and that the probability of bearing a healthy child exceeds 90%. Deficient information concerning treatment with immunosuppressives and/or biologics in pregnancy must by no means indicate a risk-based termination of pregnancy [3, 4]. Nevertheless, pregnancies in women whose primary disease requires treatment with immunosuppressives and/or biologics are regarded as high-risk, thus indicating continuous monitoring for the fetuses and mothers. Such control exceeds the extent of measures provided in pregnancy passports. Additional early-stage organ screening at the 16th gestational week (GW) are therefore recommended, possibly supplemented by early-stage glucose tolerance tests in the case of cortisone intake. Multiprofessional and fine-tuned care on the part of the treating physicians is desirable for expectant mothers [5].

Detailed preconceptional counseling of women who are under immunosuppressive therapy and who wish to become pregnant is decisive for a successful gestational course. Such advice is to respond to the possible risks and complications associated with the mothers’ disease process and course of pregnancy and with the unborn child [6, 7]. Information provided to the patients regarding the common basic risks of neonates’ congenital health problems of approximately 3% and “normal” miscarriage risks in the first trimester of approximately 15% has proven to be helpful. This holds especially true should the intake of medication not be automatically considered the cause of complications in pregnancy or infants’ health problems. It seems essential to create awareness that acute exacerbations of the underlying disease during gestation harbor a risk for mothers and their children and are to be treated [8, 9]. The risk of active episodes during pregnancy is to be discussed and/or put into perspective with the mostly feared teratogenic risk associated with the immunosuppressives and/or biologicals to be taken [10].

Should therapy become necessary in pregnancy, active involvement in treatment decisions is to be endeavored on the part of the expectant mothers in terms of shared decision making. Minor uncertainties with respect to teratogenicity may already result in misinterpretations of teratogenic risks, even though no significantly elevated risk may be indicated on close inspection. Questions regarding breastfeeding [11] and vaccinations [12, 13] should also be addressed in the preconceptional setting.

## Immunosuppressives and disease-modifying antirheumatic drugs

### Apremilast

#### Pregnancy

Due to deficient data, apremilast is not to be administered during pregnancy. (EL 5, RG D)

#### Lactation

Due to insufficient data, breastfeeding under apremilast is currently not recommended. (EL 5, RG D)

Apremilast (APR) is a drug from the group of phosphodiesterase inhibitors and is approved in Austria for the treatment of moderate to severe plaque psoriasis (PP) and psoriatic arthritis (PsA). Its anti-inflammatory effects are based on the inhibition of the intracellular enzyme phosphodiesterase-4, leading to an increase in cyclic adenosine monophosphate. The result is a decreased production of pro-inflammatory cytokines, including tumor necrosis factor alpha (TNF-α), interleukin (IL) 17 and IL 23. Animal studies have not indicated an increase in deformities under APR but a dose-dependent increase in miscarriages and reduced birth weight [14]. No published human data are available; however, a pregnancy registry has been established. In view of the highly limited data, APR therapy should be discontinued at least 2 days before conception and APR has been detected in the milk of lactating mice [14].

### Azathioprine/6-mercaptopurine

#### Pregnancy

After onset of pregnancy, remission-maintaining treatment with thiopurines, azathioprine, 6‑mercaptopurine, may be continued over the entire course of pregnancy. (EL 2, RG B)

#### Lactation

Breastfeeding is compatible with azathioprine and/or 6‑mercaptopurine therapy. (EL 3, RG B)

As azathioprine (AZA) passes the placenta, metabolites, especially the pharmacologically active 6‑thioguanine nucleotide (6-TGN) but barely 6‑methyl mercaptopurine (6-MMP), have been detected in umbilical cord blood [15]; however, children’s measured metabolite levels have shown to be clearly lower than those of their mothers. Moreover, a recent study has demonstrated statistically significant decreases in 6‑TGN levels and increases in 6‑MMP levels in expectant mothers, with no evidence of myelotoxicity or hepatotoxicity [16]. Anemia has been identified at birth in approximately 60% of the neonates, yet the Apgar scores were normal and there was no evidence of congenital malformations (CM). These effects proved to be reversible and the anemia described has not yet been reproduced in subsequent studies. A rise in thiopurine S‑methyltransferase activity during pregnancy may explain the decrease in 6‑TGN and increase in 6‑MMP, as hormonal changes in pregnancy may influence enzymes regulating the breakdown of medications [17, 18].

There have been two meta-analyses that substantiated that AZA and/or 6‑mercaptopurine (6-MP) only pose a minimal risk for fetuses [19, 20]. No differences in CM or low birth weight (<2500 g) were identified in the first analysis in comparisons between pregnant subjects given AZA or 6‑MP and those not given thiopurine treatment. A significant difference in CM and low birth weight was only shown in comparison with the overall population. The second meta-analysis found no evidence of low birth weight or CM [20]. In both publications, the increased risk of preterm delivery (prior to the 37th GW) during AZA and/or 6‑MP intake was primarily seen in connection with disease activity and not considered to be drug-related. Nevertheless, a slight risk of premature births cannot be excluded under thiopurines. Thus, a Swedish study has suggested that thiopurines elevate the risk of preterm delivery in patients with stable inflammatory bowel disease (IBD) (adjusted odds ratio, aOR, 2.41; 95% confidence interval, CI, 1.05–5.51) and active IBD (aOR, 4.90; 95% CI, 2.76–8.69) [8]. In turn, the most recent investigation, a prospective cohort study with 309 IBD patients, showed no association between mothers’ thiopurine intake (35% of pregnancies) during gestation and increased risks of spontaneous abortion, “poor” birth results, or more frequent disorders among children within their first year of life [21]. Thus, gastroenterological considerations serve to recommend continuing remission-maintaining thiopurine treatment during pregnancy [22]. The European League Against Rheumatism (EULAR) also sees no reason not to continue thiopurine treatment but has limited doses to a maximum of 2 mg/kg/body weight [23]. Due to the slow onset of effect and the risk of bone marrow suppression and pancreatitis, initiation of treatment with thiopurines during gestation is discouraged [22].

Over an observation period of 3.8 years, a prospective study in 30 children whose mothers had taken thiopurines during pregnancy and/or breastfeeding found no evidence of physical or psychosocial developmental disorders, immunodeficiencies or increased risks of infection compared to a normative control group [24]. The US Food and Drug Administration (FDA) adverse event reporting system has currently also received no reports indicating that thiopurines alone or in combination with TNF-α blockers are associated with an increased risk for mothers and their infants [25]. Finally, having published the outcomes of 797 pregnancies in abstracts only, the Pregnancy in Inflammatory Bowel Disease and Neonatal Outcomes (PIANO) registry found no elevated risks of spontaneous miscarriages, CM, preterm deliveries, intrauterine growth disorders, abnormal development or cesarean sections under thiopurines [26]; however, a higher infection rate was described in babies aged 9–12 months in the combination group (thiopurines and TNF-α blockers) compared to non-exposed children.

Within the first 4 h following intake, 6‑MP is particularly detectable in breast milk, whereas the quantity assessed corresponds to less than 1% of the maternal doses [27]. The authors therefore recommend expressing and discarding milk within 4 h after intake of the substance; however, it should be noted that this is an additional safety measure, as neither 6‑MP, 6‑TGN nor 6‑MMP have so far been detected in the blood of breastfed infants, nor is there clinical or hematological evidence of immunosuppression [28, 29]. No increase in risk of infections was observed in 15 children whose mothers had taken AZA in pregnancy and the lactation period compared to non-exposed subjects [30]. These children also showed normal courses of physical and mental development.

### Cyclophosphamide

#### Pregnancy

Cyclophosphamide is teratogenic and is to be discontinued 3 months prior to planned pregnancy. In the presence of mothers’ severe or life-threatening medical conditions, and subsequent to failure of other treatments, its administration may be considered in the second and third trimesters. (EL 2, RG C)

#### Lactation

Cyclophosphamide must not be administered in the lactation period. (EL 4, RG D)

Cyclophosphamide (CP) is a potent immunosuppressive with embryotoxic and teratogenic properties and should be discontinued 3 months prior to planned pregnancy. Accordingly, available data is limited to two cohort studies and case reports, including oncological patients and yielding a significantly increased risk for both miscarriages (OR 25 5) [31] and CM (>26%) [32, 33]. Administration of CP may only be considered in the second and third trimesters in the presence of severe (life-threatening or organ-threatening) disorders in mothers and the absence of treatment alternatives, always following strict indications. It crosses into the milk and, as an alkylating agent, may lead to bone marrow depression in neonates. It is therefore contraindicated in the lactation period.

### Cyclosporine A, tacrolimus

#### Pregnancy

Following strict indications, cyclosporine A and tacrolimus may be administered in pregnancy. (EL 2, RG B)

#### Lactation

Breastfeeding seems to be compatible with cyclosporine A and tacrolimus treatment. (EL 3, RG C)

Cyclosporine A (CsA) and tacrolimus (TAC) are second-line agents used to treat acute and severe courses of ulcerative colitis (UC), systemic lupus erythematosus, pyoderma gangrenosum and psoriasis; however, most data pertaining to gestation and lactation derive from patients treated with these substances on account of organ transplantations. Observations have become available with respect to more than 1100 pregnancies under CsA and more than 500 under TAC [23]. Increased rates of preterm deliveries and decreased birth weight were identified with both agents, which, however, may have been facilitated by the patients’ primary disorder. No elevated rates of deformities were observed under these treatments [23]. Higher rates of eclampsia and hypertension were seen in patients under CsA and of gestational diabetes in those under TAC. Cases of temporary renal failure and hyperkalemia were identified among neonates whose mothers had been given TAC during pregnancy. In line with the recommendations issued by EULAR and the Embryotox center in Berlin, and in appropriate indications, CsA and TAC may be prescribed during gestation. Patients stably medicated with CsA or TAC should not switch and level measurements are recommended.

Data on children breastfed under these treatments are available for 76 cases under CsA and 154 cases under TAC [23]. In the majority of infants, no or merely subtherapeutic levels have been detected and no side effects have been observed. The EULAR and Embryotox recommendations thus consider breastfeeding under these medications as feasible. Breastfeeding is fully possible under topical TAC but the substance must not be topically applied to the mamilla.

### Leflunomide

#### Pregnancy

Due to insufficient data, leflunomide should be discontinued 2 years prior to planned pregnancy. In any case, in women wishing to conceive or in unintended pregnancies, wash-out procedures with cholestyramine are recommended. (EL 2, RG C)

#### Lactation

Due to insufficient data, breastfeeding cannot be currently recommended under leflunomide. (EL 4, RG C)

The effect of leflunomide (LEF) is based on the inhibition of dihydroorotate dehydrogenase, pyrimidine nucleotide synthesis and protein tyrosine kinases. Animal experiments with rodents have shown the drug to be embryotoxic and teratogenic but with no corresponding CM pattern in humans. Its half-life is long and LEF can be detected in tissues up to 2 years following discontinuation, where it has an abortion-inducing effect.

A total of three studies have investigated pregnancy outcomes under LEF, both in the preconceptional setting and into the first trimester, and found no increased risk of CM. In most cases, however, wash-out procedures with cholestyramine had been carried out [34–36]. For this reason, these definitely promising data have not yet resulted in a modified assessment of LEF with respect to its application in pregnancy.

The data are similar in neurology, in the framework of which teriflunomide, the active metabolite of LEF, is applied in multiple sclerosis therapy and in which no typical CM patterns have been seen in relevant pregnancy reports [37]. Even though the substance has so far not been proven to be a major human teratogen, its application in gestation is contraindicated due to insufficient data. It is recommended to discontinue the medication 2 years prior to planned pregnancy. In women desiring to have children or in cases of unintended pregnancy, wash-out procedures with cholestyramine (8 g 3 × daily for 11 days) are recommended within 2 years after discontinuation. The objective is a negative LEF level (<0.02 g/l) in 2 level determinations at an interval of 14 days.

Due to insufficient data and the long half-life of LEF, its application is not recommended in the lactation period [23, 38].

### Methotrexate

#### Pregnancy

Methotrexate is teratogenic and must not be taken in pregnancy. Methotrexate therapy is to be discontinued 3 months prior to planned pregnancy. (EL 2, RG B).

#### Lactation

Methotrexate must not be taken while breastfeeding. (EL 2, RG B)

The folic acid antagonist methotrexate (MTX) may have a teratogenic effect, particularly in high doses, if applied during pregnancy. Given in the first trimester, high-dose MTX may result in typical embryopathy with craniofacial anomalies, anomalies of the central nervous system or extremity malformations. The MTX is applied in various doses in various indications. Thus, individual high doses of MTX, such as 1 mg/kg bw or 50 mg/m^2^, may already lead to miscarriages due to inhibition of DNA synthesis. Historically, this property was used for elective, medically induced abortion. Low doses (5–25 mg/week) are commonly applied to treat chronic inflammatory arthropathies, systemic rheumatic disorders and IBD. Only a few cases of MTX embryopathies following low-dose treatment have been reported in the literature.

A prospective multicenter cohort study [39] investigated the outcomes of 324 pregnancies in patients treated with low-dose MTX (≤30 mg/week). MTX was given to 136 patients within 12 weeks pre-conception and to 188 post-conception [39]. The pregnancies were compared with a disease-matched and a healthy cohort. Neither the risk of spontaneous abortion nor of severe CM (3.5%) was aggravated in the pre-conception cohort under low-dose MTX. The cumulative incidence of spontaneous abortion was significantly higher (42%) in the post-conception cohort than in either control group, as was the risk of severe CM (6.6% vs. 3.6% and 2.9%, respectively). There were seven children with large CM, yet none showed typical MTX-induced embryopathies [39].

A study by Martin et al. explored the outcomes of eight pregnancies in patients receiving low-dose MTX. This case series yielded one typical MTX embryopathy [40]. Other studies have found no increase in CM rates following low-dose MTX exposure in the first trimester [41]. Therefore, it is generally recommended to discontinue MTX treatment 3 months prior to planned conception [23]. Treatment discontinuation is to be accompanied by high-level folic acid substitution (5 mg/day), sustained until the end of the first trimester. The necessary minimum period between discontinuation and conception remains a matter of controversy. Based on current data, and under certain circumstances, an MTX-free interval of only 1 month may be acceptable [39]. MTX crosses into breast milk, at least in traces, while the concentration of MTX in the milk is less than 10% of that in plasma [42]. Nevertheless, breastfeeding should be refrained from under MTX [43].

### Mycophenolate mofetil

#### Pregnancy

Mycophenolic acid is a teratogenic agent and must not be taken during pregnancy. Treatment with mycophenolic acid should be discontinued 6 weeks prior to planned pregnancy. (EL 2, RG B)

#### Lactation

Due to insufficient data, breastfeeding under mycophenolic acid is currently not recommended. (EL 2, RG B)

Mycophenolic acid is a potent, selective and reversible inhibitor of inosine monophosphate dehydrogenase, which blocks the synthesis of guanine-containing nucleotides. Its half-life is 12 h following oral intake of mycophenolate sodium and 16–18 h following mycophenolate mofetil (MMF) administration. Its molecular weight is low and it thus may pass the placental barrier. In conclusion, MMF intake during pregnancy is associated with an increased teratogenic risk [44]. Hoeltzenbein et al. published the results of a prospective European multicenter cohort study with 57 pregnancies under MMF and identified a 45% cumulative incidence of spontaneous abortions and 26% risk of severe CM [45]. In addition, pregnancies were electively terminated in 12 patients, in 2 of them termination was based on evidence of multiple fetal malformations. Among the 29 live births, 6 severe malformations were detected, e. g. auditory canal atresia, tracheoesophageal atresia, hydronephrosis and atrial septal defect. A nationwide cohort study in the UK described the course of pregnancies in patients following organ transplantation [46]. Of these patients nine received MMF, amongst whom “poor fetal outcomes” were reported in seven patients, including spontaneous abortions, birth weights below 1500 g and CM. The European Medicines Agency (EMA) thus had a press release issued to point to the aggravated teratogenic risk associated with mycophenolic acid, especially when taken by men prior to conception [47].

### Tofacitinib

#### Pregnancy

Due to insufficient data, tofacitinib should be discontinued at least 6 weeks prior to planned pregnancy. (EL 4, RG C)

#### Lactation

Due to insufficient data, breastfeeding under tofacitinib cannot currently be recommended. (EL 5, RG D)

Tofacitinib (TOF) is an oral Janus kinase inhibitor approved for the treatment of rheumatoid arthritis (RA). Data on the influence of TOF in expectant mothers or its crossing into breast milk are scarce. Pregnant women have been excluded from randomized controlled approval studies, as the small-molecule substance may possibly pass to their fetuses. In spite of the compulsory application of effective contraceptive methods, pregnancies did occur in those investigations and required discontinuation of treatment and follow-up. An assessment of 47 pregnancies (33 under TOF monotherapy and 13 under TOF and MTX combination therapy) yielded no increased risk of deformity compared to the overall population. In addition, no elevated risk potential was identified among the 25 pregnancies documented in the approval studies in UC (OCTAVE 1, 2 and open-label extension) compared to non-exposed patients [48, 49]. Teratogenic effects (cardiac, skeletal and cranial malformations), increased abortion rates and lower birth weights have been observed at substantially elevated doses in preclinical animal studies. Rat models have shown TOF to cross into maternal milk [50].

In summary, no clear recommendation regarding the administration of TOF in pregnancy and lactation is currently possible on account of the low level of experience [23]. Due to hematological side effects, it is recommended to discontinue treatment 6 weeks prior to planned pregnancy in spite of its short half-life of 3 h.

## Biologics: TNF-α inhibitors

### Infliximab, adalimumab, golimumab

#### Pregnancy

Whenever clinically indicated, anti-TNF-α antibody therapy is feasible throughout pregnancy (infliximab: EL 2, RG B; adalimumab: EL 2, RG B; golimumab: EL 4, RG C). In stable clinical remission, anti-TNF-α therapy may be discontinued at the end of the second trimester (approximately 24th GW) in order to avoid infants’ exposure, even though there is no evidence of increased risks of postpartum malformation or infection. (EL 2, RG B)

#### Lactation

As only minimal concentrations of anti-TNF-α antibodies are detected in breast milk, breastfeeding is compatible with anti-TNF-α treatment. (EL 3, RG B)

#### Infliximab

The FDA has classified the antibody biologic drugs infliximab (IFX), adalimumab (ADA) and golimumab (GOL) as category B, meaning that there is no evidence so far of aggravated risks of malformation in pregnant women under these treatments. Many studies and publications on pregnancies under IFX treatment have shown no indications of elevated malformation risks [25, 51–53].

IFX is an immunoglobulin (Ig) G1 antibody which, due to its size, practically does not cross the placenta during the first trimester, the period of organogenesis. As of the end of the second trimester, IgG1 antibodies are actively transported over the placenta and therapeutic IFX concentrations are possible in the fetus [54].

Infants having been exposed to IFX after the end of the second trimester have been seen to show markedly higher IFX levels (160%) than their mothers’ sera and subsequently delayed antibody decomposition, with detectable IFX levels up to 6 months following childbirth [55]. At birth, merely four children exposed to IFX in pregnancy have so far been shown to present with transient neutropenia [56]. There are no reports to evidence IFX-associated child immunodeficiencies or developmental disorders. Normal development of T and B cells, normal Ig concentrations and adequate vaccination responses have been observed [57, 58]. however, there is one case report on a fatal disseminated Bacillus Calmette-Guérin (BCG) infection in a child exposed to IFX in utero, who had received a tuberculosis vaccination [59]. For this reason, live attenuated vaccines (LAV) are not to be administered within the first 6 months postpartum and/or until anti-TNF-α levels are no longer detectable. In Austria, however, this currently only applies to rotavirus vaccinations, which should be postponed by at least 6 months in exposed infants. Based on these data, the European Crohn’s and Colitis Organisation (ECCO) recommended in 2015 to discontinue IFX therapy in the 22nd to 24th GW, depending on the patients’ clinical status, in order to avoid children’s exposure to the largest possible extent [12]. Data published by de Lima et al. support this approach. Discontinuation of anti-TNF-α treatment in IBD patients in remission prior to the 25th GW resulted neither in an increased risk of episodes during the rest of pregnancy nor in infusion reactions or loss of efficacy following postpartum reinitiation of IFX treatment [60].

Further long-term experience concerning the development of infants’ immune systems subsequent to IFX exposure during pregnancy is currently not available. A most recent study with children exposed to anti-TNF-α during pregnancy has indicated that responses to tetanus and *Hemophilus influenzae* B vaccines were not affected and that T‑helper cell and B cell responses were to be assumed [58].

The current recommendations thus go one step further. The Toronto Consensus endorses the continuation of remission-maintaining treatment with 5‑ASA, thiopurines or anti-TNF-α monotherapy in IBD patients during pregnancy. Likewise, decisions concerning breastfeeding should be made regardless of ongoing anti-TNF-α treatment [61].

A recent study in 80 pregnant IBD patients exposed to anti-TNF-α revealed detectable IFX and ADA levels in infants up to 12 months. The median interval until elimination from the children’s circulatory systems was 4 months for ADA and 7.3 months for IFX. Among the children of mothers under anti-TNF-α and thiopurine combination treatment, the risk of bacterial or viral infections was amplified by a factor of 2.7 compared to those under anti-TNF-α monotherapy [62]. In turn, another recent study in 841 children, including 388 (46%) exposed to anti-TNF-α during pregnancy, showed no elevated risk of infection compared to non-exposed subjects. Combination treatment with thiopurines and anti-TNF-α antibodies also yielded no evidence of increased infections at a median 4‑year follow-up [63].

The recommendation remains valid to avoid LAV within the first 6 months of life (if treatment with anti-TNF-α antibodies was continued by the end of the second trimester) or within the first year of life (with continuous therapy of this kind); however, no clinical consequences seem to result from detectable anti-TNF-α levels in children [58, 62, 63].

#### Adalimumab

Although data are scarcer regarding the tolerability and safety of ADA during pregnancy compared to IFX, there is no evidence of elevated pregnancy or malformation risks under ADA. The Organization of Teratology Information Specialists (OTIS) is currently conducting a prospective study with ADA in patients with CD and RA. An interim analysis in 167 pregnant women yielded no increase in spontaneous miscarriages, stillbirths, preterm deliveries or CM compared to non-exposed pregnant patients with CD or a healthy control group [64]. Similar results have been shown in a continuous evaluation of this study in 74 ADA-exposed pregnant women with RA [65].

As with IFX, the investigated ADA concentrations from the umbilical cord blood of exposed infants were markedly higher in comparison with their mothers’ serum levels, although ADA was obviously more rapidly eliminated than IFX from the children’s circulatory systems [62].

Neither for IFX nor for ADA do the data indicate a negative effect on children’s immune systems, although long-term investigations are lacking for ADA [58].

#### Golimumab

Golimumab (GOL) is another anti-TNF-α antibody classified by the FDA as category B in pregnancy, as are IFX and ADA. Launched later than ADA or etanercept (ETN), there are few reports on its administration during pregnancy. A study in GOL administered to gravid macaques produced no evidence of changed T or B cell populations in blood or impaired immune response among their offspring [66]. Even though GOL is assumed to be safe in pregnancy, it definitely remains the TNF-α inhibitor with the lowest amount of data in this group of patients.

There are currently no reports on breastfeeding under GOL treatment.

### Certolizumab

#### Pregnancy

Certolizumab may be applied throughout pregnancy. (EL 2, RG B)

#### Lactation

Breastfeeding is compatible with certolizumab treatment. (EL 2, RG B)

Consisting of the antigen-binding fragment of a humanized anti-TNF-α antibody and polyethylene glycol, certolizumab (CZP) is a biologic that neutralizes TNF-α. It is approved for the treatment of RA, non-radiographic and radiographic axial spondyloarthritis, and PsA.

So far two cohort studies and one case-control study have investigated the safety of CZP in pregnancy and lactation, including 243 prospectively and 119 retrospectively evaluated pregnancies [23]. In these investigations, malformations and miscarriages were identified in 5% and 15% of the pregnant subjects, respectively, and were thus no more frequent than among the comparison groups. Likewise, no CM developed in a case series in Switzerland with 13 expectant mothers treated with CZP in the third trimester [23]; however, three patients developed infections, leading to a preterm delivery in one case. A most recently published evaluation of a safety database comprised prospectively accumulated data on more than 500 pregnancies, in which CZP was applied and the outcomes of which were known. In turn, resulting in no aggravated risk of CM, this analysis represents the largest cohort of women treated with an anti-TNF-α antibody during pregnancy [67].

These data have been corroborated by the recently published, prospective pharmacokinetic CRIB study, which assessed the placental transfer of CZP from pregnant women to their children. In total 16 participants were observed who received CZP as of the 30th GW. The study showed the CZP levels to be below the detection limit in 13 out of 14 infant blood samples immediately at birth and in all samples at weeks 4 and 8 postpartum [68]. Accordingly, rotavirus vaccinations and other LAV should be feasible until 6 months after childbirth; however, no data are available on this issue.

A total of seven cases of CZP application in the lactation period have been reported and CZP was not detected in the three analyzed breast milk samples [23, 69]. Moreover, the CRADLE study has recently been published. The primary objective of this prospective pharmacokinetic study was to determine the concentration of CZP in breast milk. The biological was administered to 16 breastfeeding mothers with chronic inflammatory diseases or CD. After at least 6 weeks of CZP treatment, milk samples were taken every second day for 2 weeks and analyzed for CZP [70]. Overall, very low concentrations of the agent were found in 60 breast milk samples and no measurable concentrations in 77 samples (56%) with <0.032 µg/ml. These findings serve to substantiate the recommendation by EULAR that CZP may be given in lactation. Based on these data, the EMA in January 2018 also consented to modifying the product information of CZP. According to label directions, CZP may be applied in pregnancy if clinically indicated and during lactation.

### Etanercept

#### Pregnancy

Etanercept may be applied until the 32nd GW and, if required, throughout pregnancy. (EL 2, RG B)

#### Lactation

Breastfeeding appears to be compatible with etanercept. (EL 4, RG D)

Etanercept (ETN) is a biologic consisting of the human TNF receptor p75 and the Fc end of IgG1. It binds to and neutralizes soluble TNF-α and TNF-β (lymphotoxin) and is approved for the treatment of RA, juvenile idiopathic arthritis (JIA), PsA, axial spondyloarthritis and PP. In addition to case series, cohort, case-control and registry studies have addressed the safety profile of ETN in pregnancy and lactation but no randomized or controlled studies. A EULAR committee summarized, evaluated and published the results of these investigations [23]. In total, 213 pregnancies were analyzed prospectively and 119 retrospectively. The rates of miscarriages and CM reported up to the 20th GW were 16% and 4%, respectively, and thus no higher than among RA cohorts having been given either no ETN or ETN prior to conception only. For example, no increased rate of teratogenic changes was observed in one prospective study in patients under ETN compared to non-RA patients and RA patients not treated with TNF inhibitors [71].

Among 130 pregnancies documented in the British Biologics Register, the rate of spontaneous abortions but not the rate of CM, was observed to be higher than in the control group of RA patients not treated with biologic agents [72]. Therefore, the British Society for Rheumatology, in its guidelines on prescribing biologics in pregnancy and breastfeeding, concluded that ETN may be applied until the end of the second trimester [38]. In a prospective cohort study covering several European countries and Australia, a 5% CM rate was identified among first trimester participants treated with TNF inhibitors, compared to a 1.5% rate among a control group not administered such treatment [73]. With a grade B recommendation, the EULAR committee concluded that ETN, due to its low degree of transplacental passage, may be given at least until the 32nd GW and, if required, throughout pregnancy. Conducted in the USA and published recently, a retrospective analysis of 2148 patients with chronic arthritis or psoriasis did not find ETN to be associated with aggravated gestational complications [74]. Miscarriages and stillbirths were not significantly more frequent in the group treated with ETN than among the normal population (31.7% vs. 30.1%).

The issue as to the safety of ETN during lactation is even more difficult to answer, as only four cases of breastfeeding mothers under ETN have been reported. In one of these cases, ETN concentrations in breast milk and the neonate’s serum were measured on days 41 through 43 postpartum, and in another, over the course of 12 weeks postpartum [75, 76]. A low concentration of ETN was detected in the milk, but not in the serum. The EULAR committee thus concluded that women may be treated with ETN during lactation [23].

## Other biologics

### Abatacept

#### Pregnancy

Due to insufficient data, application of abatacept during pregnancy should be avoided. (EL 4, RG D)

#### Lactation

Due to insufficient data, breastfeeding under abatacept cannot currently be recommended. (EL 4, RG D)

Abatacept (ABA) is a fusion protein with an Fc region and inhibits the costimulation of T lymphocytes. Combined with MTX, ABA is indicated for the treatment of active RA in adults and PsA. According to label instructions, ABA must not be used in pregnancy unless it is imperative. Women in childbearing years should apply effective contraceptive methods throughout treatment and until 14 weeks after their last ABA dose. ABA has been detected in rodent breast milk. It is not known whether it is secreted in human milk. Women should not breastfeed throughout treatment and until 14 weeks after the last ABA dose [77]. No negative effects on pregnancy have been found in animal studies. Publications from a case series and a case report included 152 pregnancies. By nature, an increased risk of abortion arises from the combination with MTX [78–80]. There are no studies with non-exposed comparison groups. Due to insufficient data, ABA should be avoided during pregnancy and also not be given in the period of lactation [23, 81].

### Anakinra

#### Pregnancy

In the absence of alternative treatments, anakinra may be applied during pregnancy. (EL 4, RG D)

#### Lactation

Due to insufficient data, breastfeeding under anakinra cannot currently be recommended. (EL 5, RG D)

Anakinra (ANA) is an IL-1 receptor antagonist indicated, in combination with MTX, to treat RA symptoms in adults. In addition, it is indicated for the treatment of cryopyrin-associated periodic syndromes in adults, adolescents, children and infants over the age of 8 months with at least 10 kg body weight. The spectrum of syndromes includes neonatal onset multisystem inflammatory disease/chronic, infantile, neurological, cutaneous and articular syndrome, Muckle-Wells syndrome and familial cold autoinflammatory syndrome [82].

Animal tests have been unable to evidence negative effects on pregnancy. Case reports and a recently published retrospective multicenter study comprise a total of approximately 40 cases. No increased risks of malformations or miscarriages were observed [23, 83–86].

In view of insufficient data, no specific statements concerning pregnancy are feasible. After failure of other treatment options, ANA is acceptable pre-conception and in pregnancy. Data on children having been breastfed while their mothers were under ANA show 10 cases among the recently published study without identified infections or developmental disorders [86].

### Belimumab

#### Pregnancy

Due to insufficient data, application of belimumab during pregnancy should be avoided. (EL 4, RG D)

#### Lactation

Due to insufficient data, breastfeeding under belimumab cannot currently be recommended. (EL 4, RG D)

Belimumab (BEL) is a human monoclonal anti-B-lymphocyte stimulator antibody that inhibits the development of B cells into Ig-secreting plasma cells. For several years it has been approved as an add-on to any ongoing oral immunosuppression for the treatment of systemic lupus erythematosus if high disease activity maintains in spite of standard treatment. No teratogenicity has been observed in animal models. Presently available human data on slightly more than 150 pregnancies derive from registries and case reports [87–89].

A large number of CM have been observed in the documented pregnancies, whereas virtually continuous comedication with other immunosuppressives should be noted as a limiting factor. Due to insufficient data, the specialist societies do not recommend this treatment in pregnancy. According to label instructions, BEL should be discontinued 4 months prior to planned pregnancy. The application of BEL during lactation is not recommended, as it is not known whether BEL crosses into breast milk.

### Rituximab

#### Pregnancy

Due to insufficient data, application of rituximab during pregnancy should be avoided. In the presence of severe disease progression and lacking treatment alternatives, its application is acceptable prior to conception or in the first trimester. (EL 4, RG D)

#### Lactation

Due to insufficient data, breastfeeding under rituximab cannot currently be recommended. (EL 4, RG D)

Rituximab (RTX) is a chimeric (murine/human) monoclonal antibody against the B‑cell surface antigen CD20. Intravenous administration leads to a rapid and sustained depletion in peripherally circulating CD20 B cells. Treatment schemes commonly include 4 weekly infusions of 375 mg/m^2^ or 2 infusions of 1000 mg in 14-day intervals (RA). As with other monoclonal antibodies, RTX is an IgG1 construct and is thus transported through the placenta as of the 13th GW facilitated by the fetal Fc receptor. Therefore, when given during the second or third trimester, umbilical cord blood levels are seen to be similar to or even higher than maternal levels. The estimated median half-life of RTX is approximately 20 days; however, peripheral B cells remain decreased for 6 months following infusion. The B cell neogenesis is highly heterogeneous and may even be absent for years in a small percentage of patients [90–93].

Data concerning pregnancy are available from a registry, the Rituximab Global Drug Safety Database with approximately 250 cases and from case reports of patients presenting with malignant and autoimmune disorders. There are no studies involving non-exposed comparison groups. In part, no precise statements are possible regarding the times of application.

An analysis of data from the database has yielded 153 cases with known pregnancy outcomes. About 50% of the patients were simultaneously treated with other and in part cytotoxic agents, which may explain the high percentages of spontaneous miscarriages (22%), abortions (18%) and preterm deliveries (24%). Overall, no increased rate of CM was identified (2.2%, *n* = 2). At birth hematological abnormalities including neutropenia and B cell depletions were seen in 11 infants; however, most of these changes were mild and transient, and spontaneous recovery was observed in most within weeks or months. There have been four cases of neonatal infections described (fever without a focus, chorioamnionitis, bronchiolitis and vertical transmission of cytomegalovirus). None of these infections was associated with cytopenia among the infants [94].

Due to the high rate of spontaneous miscarriages and induced abortions in the available data, a specific statement regarding pregnancy outcomes is possible to a limited extent only. The data have shown no evidence of teratogenic effects associated with RTX; however, administration in the second or third trimester has frequently resulted in reduced B cells among neonates [91, 92].

In planned pregnancies, it is thus recommended to preferably switch to another treatment. According to label instructions, RTX should be discontinued 12 months prior to planned pregnancy; however, in the presence of severe disease progression and lacking treatment alternatives, RTX treatment is acceptable prior to conception or at the beginning of the first trimester. Neonates are to be monitored with respect to infections. As there are no data concerning the period of lactation, breastfeeding is not recommended [23, 38].

### Secukinumab

#### Pregnancy

Due to insufficient data, application of secukinumab during pregnancy should be avoided. (EL 5, RG D)

#### Lactation

Due to insufficient data, breastfeeding under secukinumab cannot currently be recommended. (EL 5, RG D)

Secukinumab (SEC) is a human monoclonal anti-IL-17A antibody that also belongs to the IgG1 class. In Austria, SEC is approved to treat moderate to severe PP, PsA and ankylosing spondylitis. No published data on the application of SEC during conception or pregnancy are available. SEC has shown no negative effects on fertility in murine and simian models, and no teratogenicity has been observed [95]. In view of insufficient data, the specialist societies recommend discontinuing SEC prior to planned pregnancy. According to label instructions, SEC should be discontinued 5 months prior to planned pregnancy.

It is not known whether SEC crosses into breast milk. Its application in the period of lactation is thus not recommended.

### Tocilizumab

#### Pregnancy

Due to insufficient data, application of tocilizumab during pregnancy should be avoided. (EL 4, RG D)

#### Lactation

Due to insufficient data, breastfeeding under tocilizumab cannot currently be recommended. (EL 4, RG D)

Tocilizumab (TCZ) is a humanized monoclonal antibody (IgG) against the IL-6 receptor. It is approved for the treatment of RA and, in children above 2 years of age, for systemic JIA (Still’s disease) and polyarticular JIA.

Along with animal models, the available case reports and case series have yielded no evidence of teratogenic and/or mutagenic effects or an impact on fertility [96, 97]. An animal study showed an increased risk of spontaneous miscarriages and embryonic/fetal mortality in high doses [98].

Due to insufficient data, the application of TCZ in pregnancy is not recommended and, according to label instruction should be discontinued 3 months prior to planned pregnancy; however, treatment may be continued following strict indication criteria and risk-benefit assessments. It should be noted that TCZ, as an IgG antibody, is actively transported over the placenta. Should TCZ be applied after the 16th GW, the following postpartum consequences are possible in infants over an unknown period of time: neutropenia, lack of increase in C‑reactive protein in infections, high IL-6 levels and elevated liver values. The LAV should not be given over the first 6 months after childbirth.

As a large molecule, TCZ is not expected to cross into breast milk and be intestinally ingested by mature infants; however, no data on breastfeeding under TCZ are available and no corresponding recommendation is thus possible.

### Ustekinumab

#### Pregnancy

Due to insufficient data, application of ustekinumab during pregnancy should be avoided. (EL 4, RG D)

#### Lactation

Due to insufficient data, breastfeeding under ustekinumab cannot currently be recommended. (EL 5, RG D)

Ustekinumab (UST) is an IgG1κ human antibody, which binds to the p‑40 subunit of IL-12 and IL-23. Within the European Union it is approved for the treatment of psoriasis, PsA and CD [99–101].

Martin et al. carried out a study with UST in gravid macaques given doses that were up to 45 times higher than that applied in humans. No evidence of embryotoxic or teratogenic effects of UST was identified before or up to 6 months after birth [101]. UST has been detected in fetal serum after the 100th day of gestation until 120 days postpartum [102, 103].

Lebwohl et al. reported on 31 pregnancies in an analysis of pooled data from phase 2 and phase 3 UST studies (phase 2, PHOENIX 1, PHOENIX 2) [104]. No fetal malformations/abnormalities or miscarriages occurred in these pregnancies. Moreover, individual case reports and case series have been published on the issue of UST and pregnancy [105–110]. A total of 11 pregnancies included 9 cases of healthy neonates, 1 ongoing pregnancy and 1 spontaneous miscarriage [105]. Due to insufficient data, no specific statement regarding pregnancy is possible. According to label instructions, UST should be discontinued 15 weeks prior to planned pregnancy.

The concentration of UST detected in macaque breast milk was found to be 1000 times lower than human maternal serum [101]. Martin et al. also detected UST in neonates’ serum. The authors suspected a transplacental transfer of UST to be causative, as the UST serum concentrations decreased during the period of lactation.

There are currently no study data to evidence whether UST passes into human breast milk [111].

### Vedolizumab

#### Pregnancy

Due to insufficient data, application of vedolizumab during pregnancy should be avoided; however, in the presence of severe disease progression and lacking treatment alternatives, its application is acceptable in pregnancy. (EL 4, RG C)

#### Lactation

Due to insufficient data, breastfeeding under vedolizumab cannot currently be recommended but it appears to be unproblematic. (EL 5, RG D)

Vedolizumab (VDZ) is a humanized IgG1 antibody which specifically binds to the α4β7 integrin. Its gut-selective activity thus serves to inhibit the migration of T lymphocytes into the tissue. VDZ has only been available for the treatment of CD and UC since May 2014, thus showing a limited empirically established value. In simian models, no teratogenic effects have been observed in pregnancy and no prenatal or postnatal developmental disorders within 6 months of follow-up. Low VDZ concentrations (<300 μg/l) were detected postpartum in 3 out of 11 cynomolgus monkeys’ milk, who had received 100 mg/kg VDZ every 2 weeks. No VDZ was detected in the milk of those having received 10 mg/kg [112]. In humans, an initial report has recently been published on breastfeeding patients under VDZ treatment. The antibody was detected in minimal concentrations (<1% of maternal serum concentration), with no abnormalities observed after 3.5–10 months of follow-up in children immunized according to vaccination schedules [113].

Human studies have so far described 24 pregnancies in mothers treated with VDZ [114, 115], resulting in 11 live births (including 2 preterm deliveries), 4 spontaneous miscarriages, 5 elective abortions and 4 undocumented cases. A cerebral malformation was reported in a healthy voluntary participant with a history of two miscarriages and an ectopic pregnancy. A recent post-marketing evaluation described 81 pregnancies with 4 live births, 11 spontaneous miscarriages and 66 pregnancies with as yet undocumented outcomes [114]. In another case report on 4 pregnancies under VDZ, 1 case of pneumonia in the 35th GW was described in a patient with CD given combination treatment with AZA; however, the birth outcome was unsuspicious (four mature and healthy infants without anomalies and showing normal Apgar scores) [116].
